# Endopyelotomy vs. laparoscopic pyeloplasty

**DOI:** 10.4103/0970-1591.30257

**Published:** 2007

**Authors:** Alan Shindel

**Affiliations:** Department of Surgery, Division of Urology, Washington University, St. Louis, MO, USA. E-mail: shindela@wudosis.wustl.edu

The conventional teaching in the management of ureteropelvic junction (UPJ) obstruction in poorly functional renal units is that endopyelotomy is less likely to succeed than pyeloplasty by an open or laparoscopic approach. The authors attempt to further explore the veracity of this teaching in this retrospective review of 23 patients with poorly functioning kidneys who underwent endopyelotomy (n = 23) and 15 patients who underwent laparoscopic pyeloplasty (n = 15). The authors used resolution of symptoms (if present at baseline) and improvement in renal scan split function by at least 10% as criteria for successful intervention. There was no significant change in GFR after the intervention in either group, but all of the 11 laparoscopic patients who had been symptomatic at baseline experienced relief of symptoms, whereas just 14 of the 18 (78%) symptomatic endopyelotomy patients had improvement in symptoms. There were no complications in the endopyelotomy group, but one pyeloplasty patient required a blood transfusion and three had persistently elevated drain output. The authors conclude that symptomatic relief is more common after laparoscopic pyeloplasty, but renal function remains stable in poorly functioning kidneys regardless of approach.

The study is weakened by its retrospective and nonrandomized nature. Blinding of patient and physician with regards to treatment selection is of course not possible. Validated instruments for the objective assessment of change in pain after procedure would have been of benefit. Additionally the laparoscopic series is not quite as mature, as evidenced by the much shorter term of follow-up compared to endopyelotomy (mean 12 *vs.* 28 months respectively), although previous reports have stated that most failures of either modality occur within 1 year of intervention.[1] Nevertheless, this is one of only a few reports assessing outcomes in poorly functioning renal units.

Prior papers comparing endopyelotomy to laparoscopic pyeloplasty have demonstrated similar results to those reported by these authors, with a perceived superiority of the laparoscopic approach,[2,3] particularly in severely hydronephrotic systems.[4]

A prospective and randomized trial comparing endopyelotomy with laparoscopic pyeloplasty is needed. Until such a study becomes available, patient and physician preference will continue to drive the selection of the procedure to be performed. Patients should be counseled that laparoscopic pyeloplasty appears to lead to superior improvement in symptoms compared to endopyelotomy in poorly functioning kidneys, but this superiority comes at the expense of greater patient morbidity in the form of need for longer hospitalization and greater chance of complications such as bleeding or urinary leak.
